# Unmet need for long-acting and permanent contraceptives and associated factors among married women in Southern Ethiopia

**DOI:** 10.1186/s12905-023-02294-3

**Published:** 2023-03-29

**Authors:** Helebo Abate, Tarekegn Solomon, Teshome Abuka Abebo, Legesse Tesfaye Elilo

**Affiliations:** 1 Medical service core process, Hadiya zone Health department, Hosanna, Ethiopia; 2 School of Public Health, College of Medicine and Health Science, Hawassa University, Hawassa, Ethiopia; 3 School of Public Health, College of Medicine and Health Science, Wachemo University, Hossana, Ethiopia

**Keywords:** Unmet need, Long acting and permanent, Contraceptives, Ethiopia

## Abstract

**Background:**

Long-acting and permanent methods (LAPMs) prevent women from having unintended pregnancies. Globally, unintended pregnancies, both mistimed and unwanted, occur every year. In developing countries, maternal mortality and unsafe abortions result from unintended pregnancies. This study aimed to assess the unmet need for LAPMs of contraceptives and associated factors among married women of the reproductive age group (15–49 years) in Hosanna Town, Southern Ethiopia, in 2019.

**Methods:**

A community-based, cross-sectional study was conducted from March 20 to April 15, 2019. Data were collected on 672 current married women in the reproductive age group (15–49) through face-to-face interviews using a structured questionnaire. Study participants were selected using a multi-stage sampling method. Data were entered into the computer using EpiData version 3.1 and exported to SPSS version 20 for analysis. Bivariate and multiple logistic regressions were performed to identify factors associated with the unmet need for LAPMs. An odds ratio with 95% CI was used to assess the association between the independent and dependent variables.

**Results:**

The unmet need for LAPMs for contraception in Hossana town was 234 (34.8%) (95% CI: 29.8, 39.8). Factors significantly associated with the unmet need for LAPMs of contraception were: women’s age 35–49 [AOR = 9.01, 95% CI: 4.21, 19.32]; education of women [AOR = 8.64, 95% CI: 1.65, 45.42]; lack of discussion between partners [AOR = 4.79, 95% CI: 3.11, 7.39]; lack of proper counseling for women [AOR = 2.13, 95% CI: 1.41, 3.23]; having a daily laborer occupation [AOR = 7.08, 95% CI: 2.44, 20.51]; and attitude of women toward LAPMs of contraception [AOR = 1.62, 95% CI: 1.03, 2.56].

**Conclusions:**

The unmet need for LAPMs was high in the study area. Age of women, discussions with partners, women ever counseled by health professionals, respondents’ educational status, husband’s educational status, women’s attitude toward LAPMs, and respondents’ occupational status were contibutes for high unmet need. High unmet need contributes to an unintended pregnancy and risky abortions. Proper counseling of women and women’s discussions with their husbands is fundamental areas of intervention.

## Introduction

### Background

Family planning (FP) refers to a planned action taken by a couple to limit or space the number of children that they want to have through the use of contraceptive methods [1]. Modern methods are classified as short-acting contraceptives like condom use, injectables, and pills, and long-acting contraceptive methods like implants, and copper-containing intrauterine contraceptive devices (IUCDs) [1–4]. Female sterilization and vasectomy, on the other hand, are permanent methods. Methods such as rhythm, withdrawal, and folk methods are grouped as traditional [1].

Globally, unintended pregnancies and unsafe abortions continue to be major causes of reproductive health problems and maternal deaths. Contraceptive use prevented 218 million unintended pregnancies in developing countries, which in turn prevented 55 million unplanned births, 40 million unsafe abortions, 25 million miscarriages, and 118,000 maternal deaths, resulting in 240,000 healthy years of women’s lives each year [5]. Contraceptives contribute to women’s empowerment by reducing the burden of excess childbearing, reducing poverty, and reducing 32% of maternal deaths and 10% of childhood mortality [5, 6].

Worldwide, 13% of married women use long-acting contraceptives, but an estimated 80 million unintended pregnancies occur every year, which are both mistimed and unwanted. Among these, approximately 280,000 women die while pregnant or giving birth every year, and 99% of deaths occurred in Sub-Saharan Africa (SSA) [7, 8]. Ethiopia is one of six countries that contribute to about 50% of maternal deaths, along with India, Nigeria, Pakistan, Afghanistan, and the Congo, with 676 maternal deaths per 100,000 live births [8].

Unmet need for FP is defined as the percentage of all fertile women who are married or living in a union and thus presumed to be sexually active but are not using any method of contraception, either because they do not want to have more children, want to postpone their next birth for at least two more years, or do not know when or if they want another child [9]. Pregnant women if their pregnancy was mistimed or unwanted, amenorrhoeic women who are not using FP and whose last birth were mistimed or unwanted are considered to have an unmet need for FP. Women who are currently using an FP method are said to have a met need for FP Pregnant women whose pregnancy was mistimed or unwanted, and amenorrheic women who are not using FP and whose last births were mistimed or unwanted, are considered to have an unmet need for FP. Women who are currently using an FP method are said to have a met need for FP [1, 5, 10].

The level of unmet need for FP was much higher (24%) in SSA, which was double the world average in 2015 [7, 11, 12]. In Ethiopia, the level of unmet need for contraception was 34%, 29%, and 22% in 2005, 2011, and 2016, respectively [1, 7, 13]. Ethiopia’s demographic and health survey (EDHS) indicated that the prevalence of LAPMs (3.9%) remained low as compared to short-acting methods in 2011 [7].

LAPMs are by far the most effective methods of modern contraception available, and they are very safe and convenient [14]. The contraceptive failure rate among participants using pills was 4.55 per 100 participant-years, as compared to 0.27 participants using long-acting reversible contraceptive methods [15].

Several factors contribute to the unmet need for LAPMs of contraception, which include the following: lack of availability of trained providers; lack of commodities and supplies; and lack of adequate FP counseling [7, 16]. Fear of social disapproval or partners’ objections, worries of side effects, health concerns Fear of social disapproval or partners’ objections, worries about side effects, health concerns [17], and a lack of knowledge about contraceptive options and their use were barriers to the utilization of LAPMs [18]. However, no research evidence exists to assess the level of unmet need for LAPMs and related factors in the study area based on our knowledge.

The level of unmet need for LAPMs was 9.4% in Goba town and 16.4% in Debra Markos [19, 20]. Data from EDHS shows an unmet need of 22% for married women and the prevalence of LAPMs in Ethiopia at 10.3%. But in the region where Hossana Town is found, i.e., in the South Nations National Peoples Region (SNNPR), the unmet need for modern FP was 21%, and the prevalence of LAPMs was about 10.2% [1]. Even though the prevalence of LAPMs is nearly equal to the national level, it remains low in the region, which in turn contributes a lot to high unmet needs. According to the Hossana Town Health Office report of 2017/18, the FP service coverage was 37% and the extent of LAPMs was 10%, but there was no clear data about the prevalence of LAPMs [21]. Therefore, this study was used to assess the level of unmet need for LAPMs and their determinant factors among FP users in Hossana Town.

## Methods and materials

### Study area and period

The study was conducted in Hossana town, Hadiya zone, from March 20 to April 15, 2019. Hadiya Zone is one of the central zones in the South Nations, Nationalities, and Peoples Regional (SNNPR) State. Hossana town is the capital city of the Hadiya Zone. The town is located 230 km away from Addis Ababa and 185 km from the capital of the region in southern Ethiopia and is bordered by Lemo woreda in all directions. According to the Central Statistics Agency’s estimation of 2018 G.C., the total population of the town was approximately 107,843, of which 55,000 were women. Out of the total females, women of childbearing age were expected to be 25,127, and the number of households in the town was estimated to be 21,947 [21, 22]. Currently, four public health institutions are providing modern contraceptive methods services in the town. Among these, one is a university hospital, and the others are health centers. Administratively, the town is divided into 3 sub-cities: Setchduna, Gofarmeda, and Addisketema. The sub-cities are further divided into eight kebeles (the lower administrative level in the region): Betel, Setchduna, Arada, Bobicho, Jelonaremo, Heto, Melamba, and Lichamba [21].

### Study design

A community-based cross-sectional study was conducted.

### Source and study population

All married women of reproductive age (15–49 years) who permanently live in Hossana town. All married women of reproductive age (15–49 years) live in randomly selected kebeles in Hossana town.

### Inclusion and exclusion criteria

Those who were currently married, sexually active, fecund, and had lived in Hosanna Town for at least six months were included. Those women who were seriously sick and unable to respond were excluded from the study.

### Sample size determination and sampling techniques

#### Sample size determination

The minimum required sample size was determined by using the formula for estimation of single population proportion with the assumption of a 95% confidence level, a margin of error of 5%, and the prevalence of unmet need for LAPMs among married women in Debre Markos town in the Amhara region (32.9%) [20], and a design effect (DE) of 2. To compensate for the non-response rate, 10% was added. Therefore, for a population size greater than 10,000, n is calculated by using the formula,

$$n = (\frac{{z{{\left( {\frac{\alpha }{2}} \right)}^2}*p\left( {1 - q} \right)}}{{{d^2}}})*DE$$

Where.

- n = desired sample size.
- Z α/_2_ = 1.96 (value of Ζ at α 0.05 or critical value for normal distribution at 95% CI).
- p = estimated proportion of unmet need for LAPMs (0.329).
- q = 1-p (0.671).
- d = margin of error (0.05).
- DE = design effect (2).

Therefore, the desired total sample size (n) was 678 plus 10% (nonresponse rate) = 746.

### Sampling techniques

**Fig. 1:**
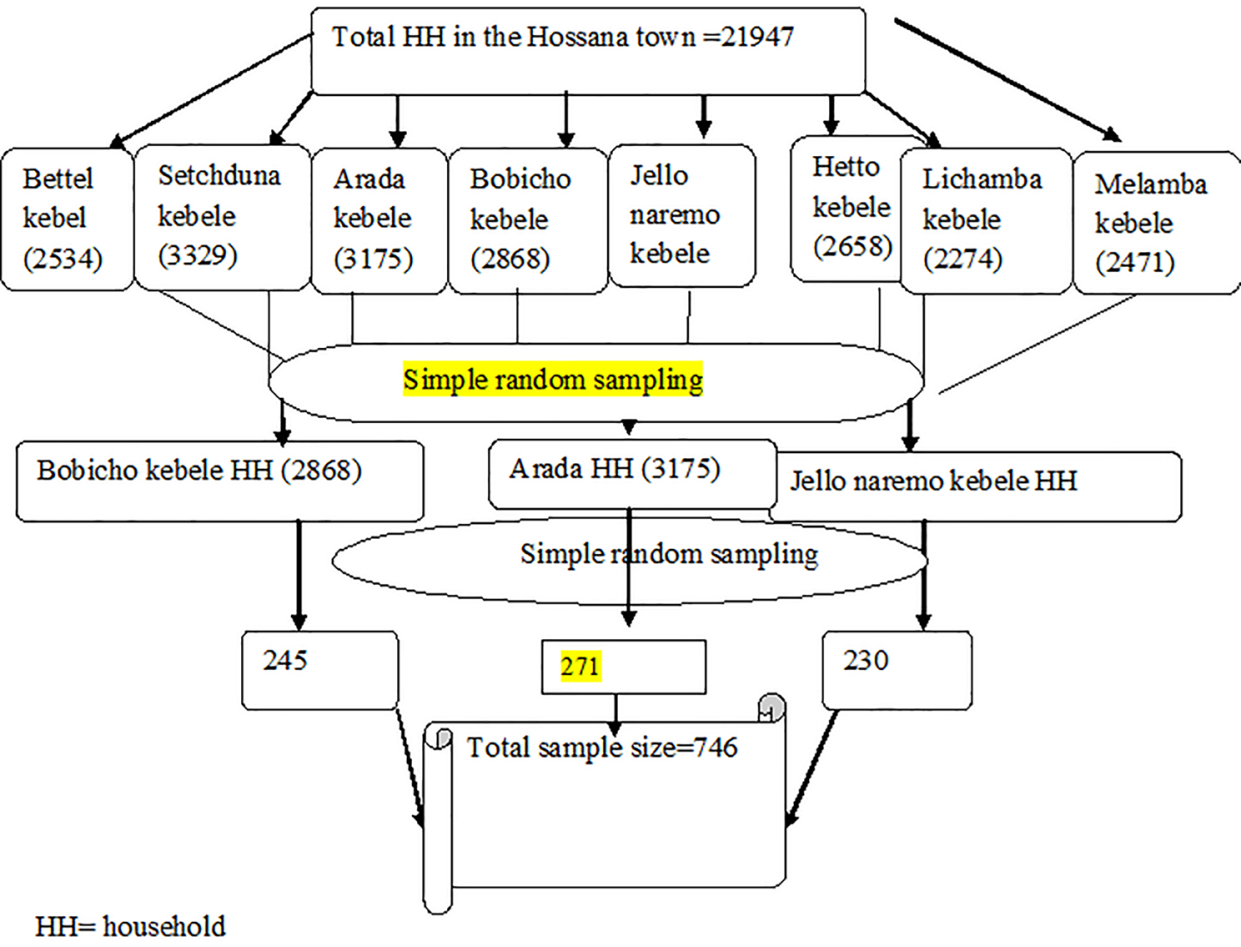
Schematic presentation of sampling procedure of married women of reproductive age in Hossana town, Southern Ethiopia

A multi-stage sampling technique was employed to select sampling units. Out of eight kebeles, three kebele (one-third of the kebeles) were selected from Hossana town by a simple random method. To get a sampling frame, a census was done in the selected kebeles based on the age and marital status of the women. During the census, eligible houses were given a house identification number. Finally, eligible married women (15–49 years) were interviewed from each kebele in selected households (Fig. 1).

### Data collection tools and techniques

The tools used for data collection were initially prepared in English and translated to the local language, Hadiyyissa, and back to English by language experts to confirm their consistency. The questions included in the questionnaire were adapted and prepared by reviewing different related literature and identifying variables to be measured [1, 23, 24]. A face-to-face interview was used to collect data. The questionnaire contains structured, closed-ended questions to collect quantitative data on the level unmet need for LAPMs and its associated factors. Six data collectors and two supervisors were recruited for data collection. The training was given to data collectors by the principal researcher before data collection. The filled tools were collected, and the consistency and completeness of the tools were checked daily by supervisors and the principal investigator until data collection ended. A pre-test of the tools was done on 5% of the total sample size at Fonko Town to familiarize interviewers with the tools, identify ambiguity, confirm the consistency of the tools, and modify any unclear parts of the tools before the actual collection of data.

### Data processing, analysis, and interpretation

The data were checked for completeness, cleaned, coded, and entered into EpiData version 3.1, then exported to SPSS version 20 for analysis. The descriptive data were summarized using frequencies, standard deviation (SD), mean, percentages, and text. And, graphs and tables were used for data presentation. Bivariate logistic regression analysis was done primarily to check which variables had a statistically significant association with the dependent variable and to select candidate variables with a P-value < 0.25 for multivariate logistic regressions after controlling for the possible effect of confounders. The outcome variables, the need for LAMPs, were dichotomized, with “1” being a need met and “0” being an unmet need for LAMPs. Finally, in multivariable logistic regression, the adjusted odds ratio (AOR) with its 95% confidence interval (CI) and p-value (< 0.05) were computed for variables maintained in the final model, and statistical significance with unmet need for LAPMs was declared with a p-value (< 0.05).

### Operational definitions

#### Unmet need for limiting

Includes currently married women who are fecund and not using any of the LAPMs of contraceptive or women who are not satisfied by using short-acting contraceptives but still need LAPMs of FP and who say they do not want any more children, pregnant women whose current pregnancy is unwanted or who are undecided whether they want another child. It also includes amenorrhea in women whose last birth was unwanted.

#### Unmet need for spacing

Includes currently married women who are fecund and not using LAPMs of FP; women who are not satisfied by using short-acting contraceptives but still need LAPMs of FP; and women who say they do not want to become pregnant soon after delivery or want to space for at least two years; or women who want another child but are unsure when to have the child; or pregnant women whose current pregnancy is mistimed. It also includes amenorrhea in women whose last birth was mistimed.

#### Total unmet need for LAPMs of family planning

The combination of women with an unmet need for limiting and spacing children.

#### Fecund

A woman of reproductive age who is capable of childbearing or becoming pregnant.

#### Knowledge of married women on LAPMs

Was assessed based on 10 items of knowledge questions. Every question answered correctly was awarded one mark, and zero for those answered incorrectly. Those who scored 9 (80%) and above had “good” knowledge, those who scored 6–8 (60–80%) had “moderate,“ and those who scored 0–5 (less than 60%) had “poor” knowledge about LAPMs.

#### The attitude of married women on LAPMs

was assessed based on six items of attitude questions. A five-point response Likert scale was used to assess the attitudes of married women on LAPMs. Responses ranged from (1) " strongly disagree”, (2) " disagree”, (3) “I don’t know”, (4) “agree”, and (5) “strongly agree.“ The individual response on list of six attitude related questionnaires was computed and was categorized into Positive/Favorable attitude and Negative/Favorable attitude towards LAMPs by calculating composite mean of each individual. Those who scored above the mean had positive attitude and those who scored the mean and below had negative attitude.

Principal component analysis (PCA): The household wealth status was classified as poor, medium, and rich.

### Ethical considerations

Ethical clearance was obtained from Hawassa University’s Institutional Review Board (IRB). Official permission was collected from the Hadiya zone health department and then from the Hossana town health office. In the end, informed consent had been obtained from all study subjects. Informed consent was obtained from a legally authorized representative for the vulnerable population. All study participants in the survey were told that their participation was voluntary and confidential.

## Results

### Socio-demographic, economic, and Reproductive history characteristics of the respondents

A total of 672 study participants, married women of reproductive age, were interviewed for the study making the response rate 90.1%. The mean age of the respondents was 28.2 years with a standard deviation of 5.1 years, and the median age was 28 years. The highest proportion of the respondents, 429 (63.8%), were age group 25–34 years. All respondents, 672 (100%), were married and living together, and residing in urban areas. The mean age of women at marriage was 20.96 ± 2.9 SD. The average number of pregnancies was 2.22 ± 1.4 SD, out of which, 232 (34.5%) experienced three and above pregnancies. Five hundred ninety-nine (89.1%) of the women aged 15–49 years responded that they want to have any more children in the future, of which 308 (51.4%) seek three and above children in their lifetime (Table 1).

**Table 1:** Socio-demographic and Reproductive history characteristics of married women in the reproductive age group in Hossana town, Southern Ethiopia, April 2019 (N = 672)

Variables		Number	Percent
Women’s age category	15–24	146	21.7
	25–34	429	63.8
	35–49	97	14.4
Religion	Protestant	479	71.3
	Orthodox	149	22.2
	Others*	44	6.5
Ethnicity	Hadiya	469	69.8
	Kambata	58	8.6
	Gurage	58	8.6
	Others**	87	12.9
Educational category of respondents	Can’t read and write	11	1.6
	Primary school (1–8)	267	39.7
	Secondary school (9–12)	207	30.8
	College diploma	113	16.8
	Degree and above	74	11.0
Occupation	Government Worker	161	24.0
	House Wife	318	47.3
	Merchant	101	15.0
	Nongovernmental organization	42	6.3
	Daily laborer	52	7.7
Educational status of husband	Can’t read and write	6	0.9
	Primary school (1–8)	182	27.1
	Secondary school (9–12)	168	25
	College diploma	82	12
	Degree and above	234	34.8
Wealth index	Poor	224	33.3
	Medium income	224	33.3
	Rich	224	33.3
Age at marriage	Less than 18	52	7.7
	Greater than or equal to 18	620	92.3
Ever had pregnancy	None	34	5.1
	one/two	406	60.4
	Three	123	18.3
	four and above	109	16.2
Number of children alive (n = 634)	None	2	0.3
	one/two	436	68.8
	Three	108	17.0
	four and above	88	13.9
Desire for more children	No	73	10.9
	Yes	599	89.1
Number of desired children	None	73	10.9
	one/two	291	43.3
	three and above	308	45.8
Experience of abortions	No	571	85.0
	Yes	101	15.0
Number of abortions (n = 101)	1	76	75.2
	+ 2	25	24.8

Others*- Muslim, Catholic, Others**- Amhara, Oromo, Silte

### Proportion of unmet need for LAPMs of contraceptives

**Fig. 2:**
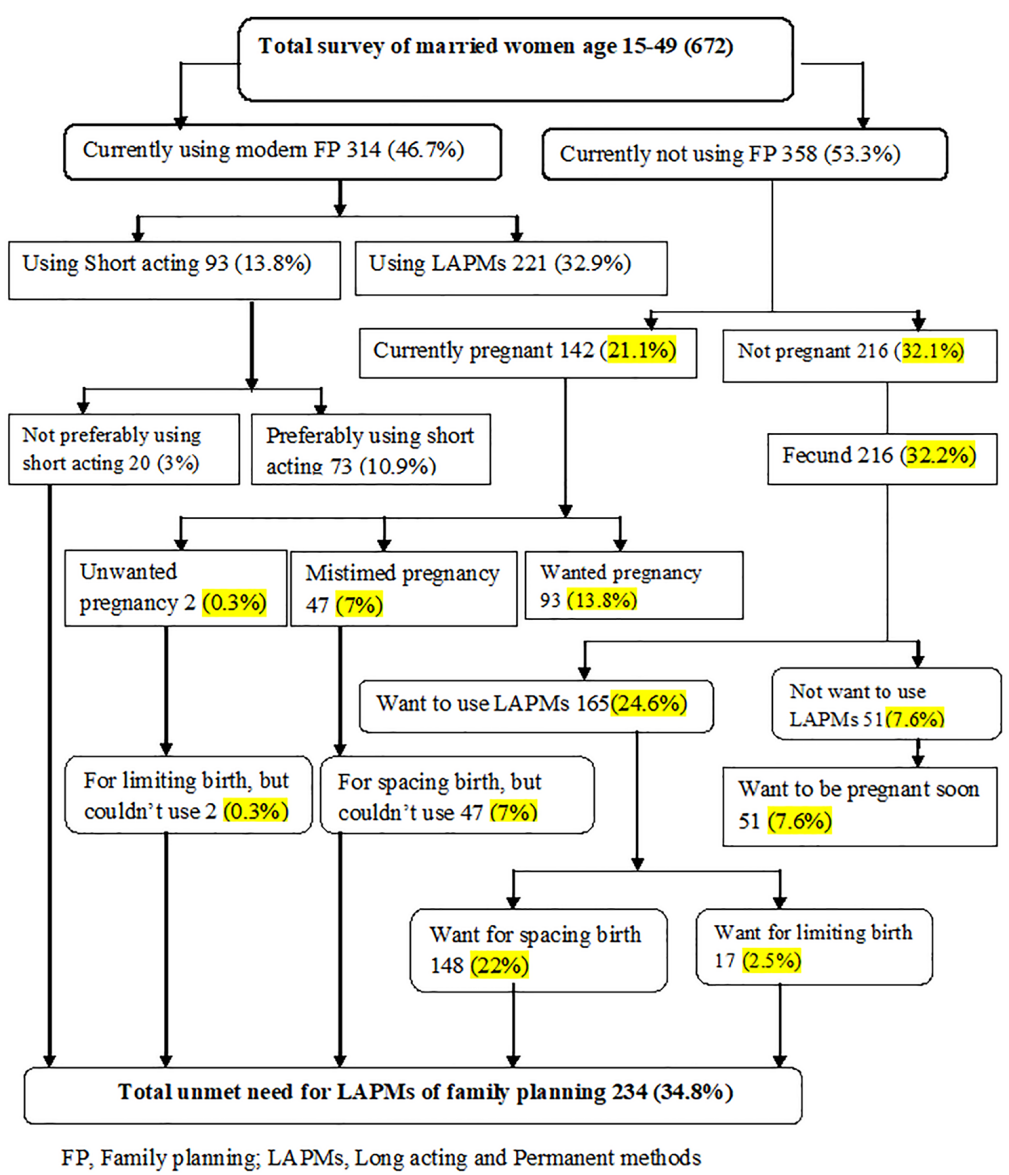
Unmet need of LAPMs of contraception among married women in the reproductive age group (15-19) in Hossana town, Hadiya zone, Southern Ethiopia, 2019 (Westoff Model)

The total unmet need for LAPMs of contraceptives in the Hosanna town was 234 34.8%) (**95% CI: 29.8, 39.8)**. From one hundred forty-two (21.1%) pregnant women, 47 (7%) complained that their pregnancies were mistimed, and 2 (0.3%) were unwanted pregnancies that had the desire to space birth for more than two years. Out of the total nonpregnant respondents, 216 (32.2%) were not using any contraceptives, of which 165 (24.6%) had the desire to use LAPMs of contraception and wants to extend their pregnancy for more than two years (Fig. 2).

### The utilization rate of family planning

**Fig. 3:**
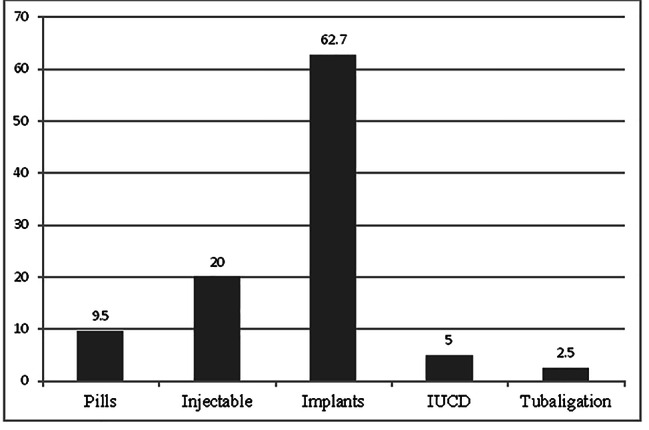
Modern contraceptive methods practiced among married women of reproductive age in Hossana town, Southern Ethiopia, April 20129

The current utilization rate of FP in the town was 314 (46.7%). The prevalence of LAPMs of contraceptives in the town was 221 (32.9%) (Fig. 2). Out of current FP users, 197 (67.2) Implants, 16 (5%) IUCD and 8 (2.5%) women were using Tubal ligation (Fig. 3).

### Source of information & discussion about LAPMs

Most of the respondents, 643 (95.7%) heard at least one LAPM of contraceptives. The major source of information was health professionals and about 392 (61.0%) of the respondents were ever counseled about LAPMs of contraception by health professionals. Four hundred fifty-four (67.6%) of the respondents discussed with their husbands/partners, of these 158 (23.5%) discussed LAPMs sometimes with their husbands/partners (Table 2).

**Table 2:** Source of information & discussion about LAPMs among women of reproductive age (15–49) in Hossana town, Southern Ethiopia, 2019 (n = 672)

Variable	Number	Percent
**Have you ever heard LAPMs**		
No	29	4.3
Yes	643	95.7
**What is the source of information (n = 643)**		
Neighbors or Relatives	96	14.9
Husband	18	2.8
Health Professionals	392	61.0
Mass media TV or Radio	130	20.2
Others	7	1.1
**Discussion with partner**		
No	218	32.4
Yes	454	67.6
**How frequently discuss (n = 454)**		
Some times	158	23.5
Once in Week	152	22.6
Once in Month	132	19.6
Once in year	12	1.8
**what is your husband’s opinion (n = 454)**		
Agree	323	71.1
I don’t know	33	7.3
Disagree	98	21.6
**Ever been counseled about LAPMs by a health professional**		
No	280	41.7
Yes	392	58.3

### Knowledge level of women about LAPMs of contraceptives

Ten knowledge LAPMs itemes were coded as poor, moderate and good based on based score of respondants. These items covered knowledge within the scope on the contraceptive mode of action, types, timing, and their uses. Those who scored between 0 and 5 (less than 60%) marks were regarded as having poor knowledge, between 6 and 8 (60-80%) as having moderate knowledge while 9(80%) and above were regarded as having good knowledge on LAPMs of contraceptives. Out of total responadents, 34.5% and 47% respondandents had good knowledge and moderate knowledge on LAPMs of contraceptives respectively (Table 3).

**Table 3:** Knowledge level on LAPMs of contraceptives among women in the reproductive age group (15–49), in Hossana town, Southern Ethiopia, April 2019

Knowledge assessment	Number	Percent
**Do you know where LAPMs of FP obtained**		
No	43	6.4
Yes	629	93.6
**Mention where it is obtained (n = 629)**		
Health Center	483	76.8
Hospital	123	19.6
Merstops International	15	2.4
Private Clinics	7	1
Others	1	0.2
**IUCD prevents pregnancy for about 3 to 12 years**		
No	261	38.8
Yes	411	61.2
**IUCD hinders sexual intercourse and decreases sexual desire**		
Yes	393	58.5
No	279	41.5
**Pregnancy can be prompt after the removal of IUCD**		
No	248	36.9
Yes	424	63.1
**Implants can prevent pregnancy for 5 years**		
No	91	13.5
Yes	581	86.5
**Implant insertion and removal need minor surgery**		
No	72	10.7
Yes	600	89.3
**pregnancy can be prompt after the removal of the implant**		
No	103	15.3
Yes	569	84.7
**Vasectomy does not have any impact on sexual intercourse**		
No	398	59.2
Yes	274	40.8
**Pregnancy is not possible after tubal ligation**		
No	187	27.8
Yes	485	72.2
**A total score of the knowledge level of respondents**		
Poor knowledge (0–5)	124	18.5
Moderate knowledge (6–8)	316	47
Good knowledge (9 and above)	232	34.5

### The attitude of women toward LAPMs of contraception

The minimum and maximum attitude scores of respondents were 6 and 30 by using a six-item attitude measuring questionnaire, respectively. 58% of respondents had a negative/unfavorable attitude toward LAPMs (Table 4).

**Table 4:** Descriptive of Scores of attitude on LAPMs of contraceptives, in Hossana town, Southern Ethiopia, April 2019

Attitude assessment	Number	Percent
**I believe that IUCD disappears in the abdomen if it inserted for ten years**		
Strongly disagree	48	7.1
Disagree	211	31.4
I don’t know	297	44.2
Agree	107	15.9
strongly agree	9	1.3
**I believe that an Implant disappears in the muscle of the hand if it inserted for three to five years**		
Strongly disagree	117	17.4
Disagree	384	57.1
I don’t know	130	19.3
Agree	41	6.1
Strongly agree	0	0
**I feel that surgery for the insertion of an implant is dangerous for me**		
Strongly disagree	45	6.7
Disagree	223	33.2
I don’t know	339	50.4
Agree	64	9.5
strongly agree	1	0.1
**I think during insertion and removal of IUCD have no privacy**		
Strongly disagree	7	1.0
Disagree	62	9.2
I don’t know	263	39.1
Agree	334	49.7
Strongly agree	6	0.9
**If use IUCD, I will suffer from severe pain during the Insertion and removal of IUCD**		
Strongly disagree	5	0.7
Disagree	76	11.3
I don’t know	334	49.7
Agree	254	37.8
Strongly agree	3	0.4
**I think that discussion about LAPMs with husbands or friends is not good for me**		
Strongly disagree	186	27.7
Disagree	180	26.8
I don’t know	23	3.4
Agree	277	41.2
strongly agree	6	0.9
Negative/ unfavorable attitude	392	58.3
Positive/favorable attitude	280	41.7

### Associated factors with unmet need for LAPMs of contraceptives at bivariate and multivariable logistic regression

In the bivariate logistic regression analysis, women’s age, respondents’ education status, husband’s educational status, occupational status, number of children alive, knowledge about LAPMs, women’s discussions with their partners, women ever counseled by health professionals, educational status, attitude toward LAPMs, and occupational status of the respondents were significantly associated with unmet need for LAPMs and candidate variables to run under multivariable logistic regression (Table 5).

**Table 5:** Factors associated with unmet need for LAPMs contraceptives at bivariate and multivariable logistic regression among married women of reproductive age in Hossana town, Southern Ethiopia, 2019

Variables	Unmet need for LAPMs		COR (95% CI)	AOR (95% CI)
	No	Yes		
Occupational status				
Government Worker	131 (81.4%)	30 (18.6%)	1	1
House Wife	183 (57.5%)	135 (42.5%)	3.2 (2.04, 5.07)	1.00 (0.42, 2.37)
Merchant	73 (73.7%)	26 (26.3%)	1.5 (0.85, 2.82)	0.45 (0.17, 1.23)
Nongovement worker	34 (81%)	8 (19%)	1.02 (0.43, 2.4)	0.50 (0.16, 1.56)
Daily laborer	17 (32.7%)	(35)67.3%	8.9 (4.45, 18.14)	7.08 (2.44, 20.51)*
Ever been counseled by health professionals				
No	154 (55%)	126 (45%)	2.2 (1.55, 2.97)	2.13 (1.41, 3.23)
Yes	284 (72.4%)	108 (27.6%)	1	1
Attitude of the respondents				
Negative attitude	238 (60.7%)	154 (39.3%)	1.6 (1.16, 2.24)	1.62 (1.03, 2.56)
Positive attitude	200 (71.4%)	80 (28.6%)	1	1
Knowledge of respondent				
Poor	74 (59.7%)	50 (40.3%)	1.4 (0.92, 2.77)	0.72 (0.37, 1.37)
Moderate	206 (65.2%)	110 (34.8)	1.14 (0.79, 1.6)	0.78 (0.48, 1.28)
Good	158 (68.1%)	74 (31.9%)	1	1
Women’s age				
15–24	105 (71.9%)	41 (28.1%)	1	1
25–34	304 (70.9%	125 (29.1%)	1.05 (0.69, 1.59)	1.30 (0.76, 2.4)
35–49	29 (29.9%)	68 (70.1%)	6 (3.14, 10.56)	9.01 (4.21, 19.32)
Educational status of respondents				
Can’t read and write	4 (36.4%)	7 (63.6%)	7.5 (1.93, 29.2)	8.64 (1.65, 45.42)
Primary school (1–8)	152 (56.9%)	115 (43.1%)	3.2 (1.73, 6.09)	1.71 (0.67, 4.35)
Secondary school (9–12)	130 (62.8%)	77 (37.2%)	2.5 (1.33, 4.84)	1.87 (0.72, 4.88)
College diploma	92 (81.4%)	21 (18.6%)	0.95 (0.46, 2.07)	0.84 (0.35, 2.01)
Degree and above	60 (81.1%)	14 (18.9%)	1	1
Husband’s educational status				
Can’t read and write	3 (50%)	3 (50%)	3.5 (0.68, 17.85)	2.24 (0.36, 13.78)
Primary school (1–8)	95 (52.2%)	87 (47.8%)	3.2 (2.09, 4.89)	2.14 (1.15, 3.98)
Secondary school (9–12)	101 (60.1%)	67 (39.9%)	2.3 (1.5, 3.59)	1.91 (1.05, 3.50)
College diploma	57 (69.5%)	25 (30.5%)	1.5 (0.87, 2.69)	1.64 (0.79, 3.38)
Degree and above	187 (77.8%)	52 (22.2%)	1	1
Discussion with husband				
No	95 (43.6%)	123 (56.4%)	4 (2.84, 5.63)	4.79 (3.11, 7.39)
Yes	343 (75.6%)	111 (24.4%)	1	1

However, after controlling the effect of other variables (confounders), in the multivariate logistic regression analysis, women’s age, women’s discussion with their partners, women ever counseled by health professionals, educational status of the respondents, educational status of the husband, attitudes of women toward LAPMs of contraception, and occupational status of the respondents were significantly associated with unmet need for LAPMs (Table 5).

Occupational status was an important predictor of unmet LAPMs of contraception. Daily laborers’ had about seven times higher unmet needs than government workers [AOR: 7.08, 95% CI: 2.44, 20.50]. Education status was an associated factor of unmet need for LAPMs of contraception, respondents who can’t read and write were found to be about nine times more likely to have had an unmet need for LAPMs of contraception than those respondents who had a degree and above levels of education [AOR: 8.64, 95% CI: 1.64, 45.42]. Husband’s educational status was also found to be an associated factor of the unmet need for LAPMs of contraception; women whose husband’s educational level was in primary school [AOR: 2.14, 95% CI: 1.16, 3.98] and secondary school [AOR: 1.91, 95% CI: 1.04, 3.5; P = 0.03] were found to be about two times more likely to have had an unmet need for LAPMs of contraception than those whose husband’s educational level was degree and above (Table 5).

## Discussion

This community-based cross-sectional study attempted to assess the unmet need for LAMPs and associated factors among married women in Hossana town. The study revealed that more than one-third (34.8%) (**95% CI: 29.8, 39.8)** of the respondents had an unmet need for LAPMs, of which 32% had an unmet need for spacing and 2.8% for limiting. It is almost comparable with the study done in Shashemene, Oromia region, and Debra-Markos, Amara region, where the unmet need for LAPMs was 33.3% and 32.9%, respectively [20, 23]. It is lower than a survey conducted in Butajira, which had an unmet need of 52.4% [25]. The reason for this might be the difference in time and study setting in which it was conducted in both rural and urban settings, as well as the general FP methods. However, this study is only for urban areas with an unmet need for LAPMs. It was higher than studies conducted in Goba (9.4%) and Debra Tabor (7.8%) [19, 26]. The reason for this might be the study design, in which it was conducted in facilities where all study participants were FP users, but this study is based on a community where study participants are not specified.

In this study, women’s age, spousal discussion about contraception, attitudes of the respondents toward LAPMs of contraception, educational status of the respondents and their partners, getting counseling from health professionals about LAPMs, and occupational status of the respondents were significantly associated with an unmet need for LAPMs of contraception.

Accordingly, women’s age between 35 and above years were almost nine times more likely to have an unmet need for LAPMs than women whose age was between 15 and 19 years [AOR = 9.01, CI: 4.21, 19.32]. This result is supported by a study done in Batu Jira Town and Debra-Markos [20, 27]. The reason for the age difference could be that older women had more children and had a greater desire to limit or space the number of pregnancies than younger women who had none or few children. This is also supported by studies done in Goba town, Oromia region, in which women who wanted more children were younger [19].

According to the study, couples who did not discuss LAPMs of contraception were nearly five times more likely to have unmet LAPM needs than couples who did. This could be because individuals who did not have a conversation were less likely to discuss the desired family size, understand how their partner feels about LAPMs, and want to utilize appropriate methods than those who had satisfied their need for the methods. Studies conducted in Goba, Shashemene, Mekele, and Debre-Tabore [19, 23, 24, 26] that showed a significant association between contraceptive use and spousal discussion of FP also support this finding.

This study also shows that women who did not receive the required counseling from healthcare workers on using LAPMs for contraception were about two times as likely to have unmet needs for LAPMs as women who did receive the appropriate consultation. Healthcare professionals’ counseling of women was one of the associated variables for the unmet need for LAPMs, according to studies conducted in Shashemene [23]. This research suggests that FP service providers should be aware of their responsibilities and held accountable for providing women with appropriate counseling and health information about LAPMs.

The woman’s occupation was another significant factor in the association between unmet need for LAPMs; it was found that respondents who were classified as daily laborers were seven times more likely to have had an unmet need for LAPMs than respondents with other occupations [AOR: 7.08, 95% CI: 2.44, 20.50]. A study conducted in northwestern Ethiopia supports our study’s findings [26].

Education of women was found to be an associated factor of unmet need for LAPMs of contraception, respondents who couldn’t read and write (illiterate) were found to be about eight times more likely to have had an unmet need for LAPMs of contraception than those respondents who had degrees and above levels of education. This finding is similar to studies done in Arbaminch, Shashemene, and Gonder [5, 23, 28]. This may be a result of the fact that educated women are more likely to know about contraceptive methods and to be more convinced when approaching service providers than women with no education. Moreover, it affects positively women’s attitudes toward contraceptive use and puts them in a position to negotiate contraceptive adoption. Furthermore, the educational status of the husband was also found to be an associated factor of the unmet need for LAPMs of contraceptives; women whose husband’s educational level was in primary school and secondary school were found to be about two times more likely to have had an unmet need for LAPMs of contraception than those whose husband’s educational level was a degree and above.

Those women who had a negative attitude towards LAPMs of FP use were nearly two times more likely to have unmet needs than women with a favorable attitude (AOR = 1.6, 95% CI: 1.03, 2.56).

## Conclusions and recommendations

In the Hossana town, current married women were found to have an unmet need for LAPMs of contraceptives was high. Women’s age, women’s discussion with their partners, women ever counselled by health professionals, educational status of the respondents, educational status of the husband, attitude of women and occupational status of the respondents affect LAPMs utilization and also contribute for high unmet need. This demonstrated that there is a chance of an unintended pregnancy and risky abortions.

### Recommendations

Concerned health managers and support organizations should put more effort into educating healthcare professionals on how to support women and convince their spouses to use LAPMs.

Strengthen behavioural change communication to increased knowledge and change attitude of women about LAMPs and about its utilazation.

It was urged that researchers carry out additional research to determine the amount and related factors of the unmet need for LAPMS of FP for all sexually active women.

### Limitations of the study

This study has a drawback in terms of not including men as study participants, so it was not comprehensive enough to represent all males. Moreover, as it is a cross-sectional study it could be difficult to establish a temporal relationship. Furthermore, this study was not triangulated by a qualitative study. This study was effect by nonresponse bias due to study participants not wanting a response, no available at home, and being unable to reach respondents during the data collection period.

## Data Availability

All the necessary data are available within the paper.

## Abbreviations

AOR: Adjusted Odds Ratio
COR: Crude Odds Ratio
DHS: Demographic Health Survey
EDHS: Ethiopian Demographic Health Survey
EMDHS: Ethiopian Min Demographic Health Survey
FP: Family Planning
HH: Household
IRB: Institutional Review Board
IUCD: Intrauterine Contraceptive Device
LAPMs: Long Acting and Permanent Methods
OR: Odds Ratio
SNNPR: South Nations Nationalities and Peoples Region
SPSS: Statistical Package for Social Science
